# Synaptic activity and strength are reflected by changes in the post-synaptic secretory pathway

**DOI:** 10.1038/s41598-020-77260-2

**Published:** 2020-11-25

**Authors:** Clara-Marie Gürth, Tal M. Dankovich, Silvio O. Rizzoli, Elisa D’Este

**Affiliations:** 1 Department of NanoBiophotonics, Max Planck Institute for Biophysical Chemistry, Am Fassberg 11, 37077 Göttingen, Germany; 2 Department of Optical Nanoscopy, Max Planck Institute for Medical Research, Jahnstr. 29, 69120 Heidelberg, Germany; 3 Institute for Neuro- and Sensory Physiology, University Medical Center Göttingen, Humboldtallee 23, 37073 Göttingen, Germany; 4 Optical Microscopy Facility, Max Planck Institute for Medical Research, Jahnstr. 29, 69120 Heidelberg, Germany

**Keywords:** Secretion, Super-resolution microscopy, Endoplasmic reticulum, Golgi, Cellular neuroscience, Spine regulation and structure

## Abstract

Neurons are highly asymmetric cells that span long distances and need to react promptly to local demands. Consequently, neuronal secretory pathway elements are distributed throughout neurites, specifically in post-synaptic compartments, to enable local protein synthesis and delivery. Whether and how changes in local synaptic activity correlate to post-synaptic secretory elements is still unclear. To assess this, we used STED nanoscopy and automated quantitative image analysis of post-synaptic markers of the endoplasmic reticulum, ER-Golgi intermediate compartment, trans-Golgi network, and spine apparatus. We found that the distribution of these proteins was dependent on pre-synaptic activity, measured as the amount of recycling vesicles. Moreover, their abundance correlated to both pre- and post-synaptic markers of synaptic strength. Overall, the results suggest that in small, low-activity synapses the secretory pathway components are tightly clustered in the synaptic area, presumably to enable rapid local responses, while bigger synapses utilise secretory machinery components from larger, more diffuse areas.

## Introduction

The secretory pathway elements support cellular functions by ensuring local delivery and post-translational modifications of secreted and transmembrane proteins. These proteins are translated directly into the endoplasmic reticulum (ER) lumen, and transported to the Golgi apparatus via the ER-Golgi intermediate compartment (ERGIC)^1,2^. Then, cargoes exit the Golgi apparatus via the trans-Golgi network (TGN), where the proteins are sorted and transported to their respective sites of action^3^. Due to their highly asymmetrical shape, size and subcellular specialisation, neurons need to organise protein synthesis along their entire volume (for reviews^4–8^). Protein processing through a secretory pathway organised exclusively in the soma would not allow a prompt supply of newly synthesised proteins to distal regions, and would not meet the specific needs of each subcellular compartment^9^. To compensate for this limitation, the protein synthesis machinery (mRNAs and ribosomes) has been reported in both pre- and post-synaptic areas^10^, and other secretory pathway elements are also widespread or asymmetrically distributed, to ensure an adequate protein supply at specific subcellular locations^11^.

The neuronal ER forms a branched network, stretching from the soma to dendrites and axonal pre-synaptic terminals, albeit it is far more abundant in dendrites than in axons^11,12^. Although it spans over long distances, the ER still remains one single membrane organelle, with a continuous lumen^13^. The ER has been found within dendritic spines, where it shows high turnover, contributes to long-term potentiation (LTP), and a specialised, smooth ER variant forms the spine apparatus (SA)^14,15^. The SA has been documented in a subset of spines and is involved in calcium storage, synaptic strength, and robust potentiation^16–20^. The SA is bound to the actin cytoskeleton of the spine by the marker and essential component synaptopodin^21–23^. This protein is required for calcium regulation and actin remodelling^18–20,24^. However, synaptopodin has also been shown to be a potential marker of SA-unrelated dynamics^15,25^.

Similar to the ER, the neuronal ERGIC is also found in the cell body and dendritic shafts, but is rarely present in spines^11,26–28^. Within the spine, ERGIC is often located in close proximity to recycling endosomes, which are similar in shape. Thus, discriminating between them unambiguously by electron microscopy is difficult^26,27^. Trafficking of ERGIC vesicles along dendrites is restricted by increased neuronal activity, indicating that this post-ER compartment can be subjected to synaptic activity-dependent regulation^29^.

Unlike the other organelles of the secretory pathway, the neuronal Golgi apparatus localises mainly around the nucleus and along the first portion of the apical or longer dendrite^30,31^. However, smaller Golgi outposts can be found in a subset of dendrites and small Golgi satellite structures have been observed throughout the entire dendritic tree, often in close association to the ERGIC^31,32^. In addition, a marker of the trans-Golgi network, TGN38, has been observed along the whole dendritic tree, including small dendrites and spines^2,33–35^. TGN38, whose synthesis can be modulated by neuronal activity, is present in vesicles that cycle between the plasma membrane and intracellular membranes, probably facilitating rapid changes in the protein composition of post-synaptic membranes^33,36^.

Synapses are plastic and functionally diverse compartments where information is passed from the pre- to the post-synaptic cell. The amplitude of the post-synaptic response, commonly referred to as the synaptic strength, can be modulated by changes at both the pre- and post-synaptic levels (for reviews see^37–42^). At the post-synapse, larger post-synaptic densities (PSD) reflect a higher number of functional receptors, and hence a stronger and more potentiated synapse^43,44^. At the pre-synaptic level, synaptic strength can be modulated, among others, by the availability of vesicles and release machinery proteins^45^. In glutamatergic neurons, pre-synaptic strength can be approximated by labelling the vesicular glutamate transporter vGLUT1^46^, which reveals the entire vesicle pool. At the same time, synaptic activity can be approximated for the pre-synaptic compartment by measuring the size of the recycling pool of vesicles, using a live labelling of the vesicular protein synaptotagmin-1 (SYT1), which specifically reveals these vesicles^47–49^. The recycling vesicles compose about 20 to 50% of all vesicles^49^. Their number is variable, and depends not only on the size of the total pool of vesicles, but also on many other parameters, as the metabolic age of the vesicles^49^. Therefore, although the last two measurements are related, they are not identical, with the vGLUT1 amounts providing only a measurement of the vesicle numbers, while the live SYT1 labelling also provides an indication of local activity.

The brief overview provided in this introduction indicates that the neuronal distribution of the secretory organelles is well studied, and that several lines of evidence connect them to synaptic dynamics. However, it is still unclear whether and how the characteristics of the synapses impact the abundance and localisation of the secretory pathway elements. Here, we used STED nanoscopy and quantitative image analysis to investigate the relationship between several secretory elements and synaptic activity. By using calreticulin, ERGIC53, TGN38 and synaptopodin as markers for the ER, ERGIC, TGN and SA, respectively, we found a positive correlation between indicators of synaptic strength and the abundance of these markers in close proximity of the synaptic area. Furthermore, a read-out of pre-synaptic activity correlated strongly with the distribution of secretory pathway elements. Therefore, these results indicate that both the quantity and position of the secretory elements is dependent on synaptic strength and activity, presumably in order to enable an optimal synaptic response.

## Results

### Secretory pathway elements are present in proximity to post-synaptic sites

**Figure 1:**
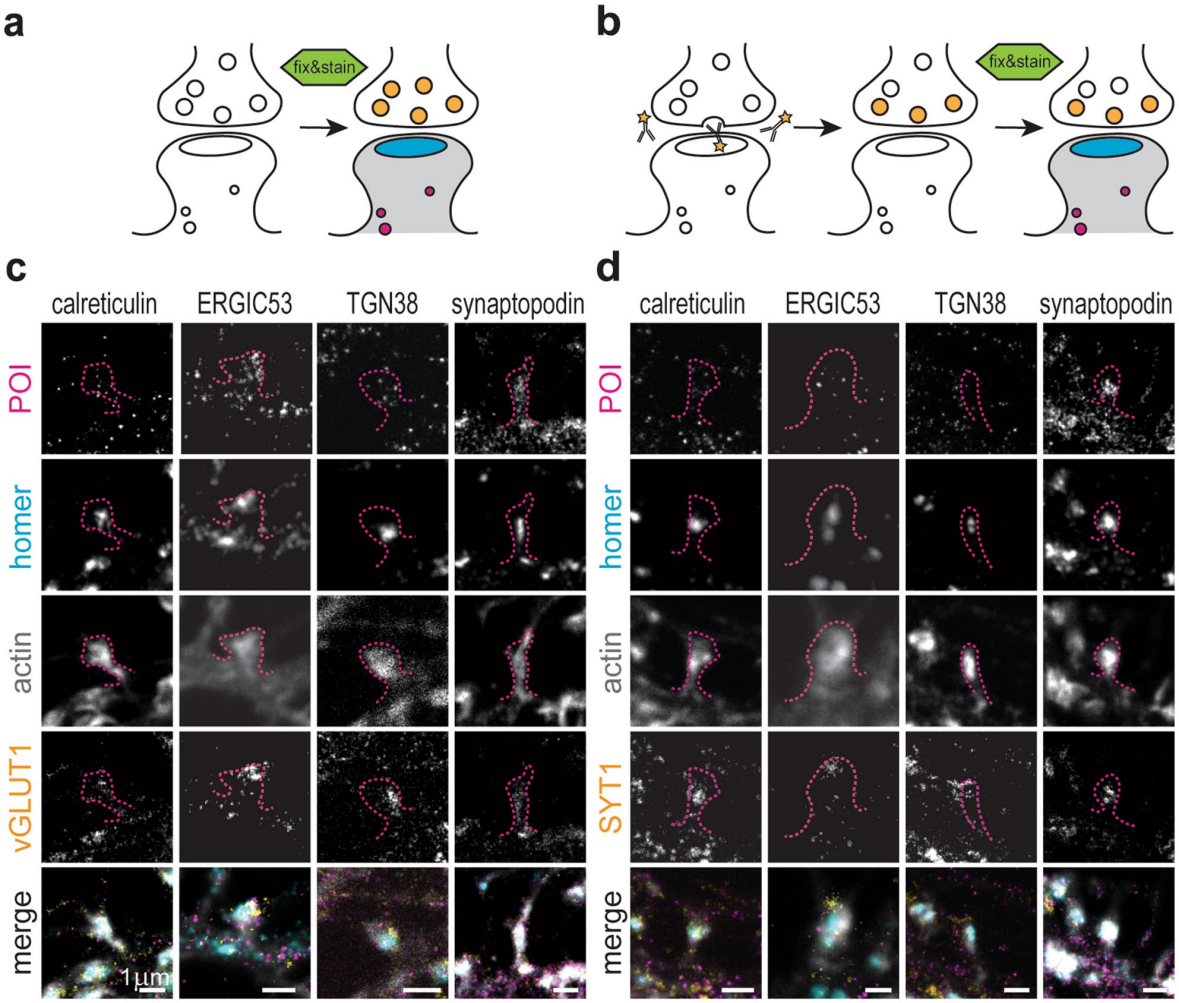
Experimental design and presence of secretory elements at post-synaptic sites. (**a**) Cells were either directly fixed and stained when visualising vGLUT1 or (**b**) incubated with an Atto647N-tagged anti-SYT1 antibody directed towards the extracellular domain of SYT1 prior to fixation. When a vesicle fuses with the pre-synaptic site, the SYT1 epitope becomes accessible to the antibody, which is incorporated by endocytosis within the recycling synaptic vesicle. Thereby, this label provides an estimate of activity at the respective synaptic boutons. (**c**,**d**) Representative STED images of individual synaptic sites in mature hippocampal neuron cultures. Samples were stained for several proteins of interest (POI: calreticulin, ERGIC53, TGN38, synaptopodin), with pre- and post-synaptic sites markers (vGLUT1 or SYT1, and homer, respectively), and phalloidin (actin, confocal). Secretory elements were found in the proximity of post-synaptic sites. Dashed lines represent the spine outline as labelled by phalloidin. Scale bars: 1 µm.

To determine whether the features of a synapse influence the local distribution and abundance of the elements of the secretory pathway, or their proximity to the synapse, we designed a high throughput experiment that enabled simultaneous super-resolution imaging of multiple proteins and parameters in cultured hippocampal neurons. The parameters selected to define synapses were the fluorescence signal intensities of the following: homer, a post-synaptic structural marker^44^; vGLUT1, a marker of the entire pre-synaptic vesicle population of excitatory nerve terminals in these cultures^46^; and live labelling of SYT1 for identifying only the actively recycling synaptic vesicles in pre-synaptic boutons^48^. While the first two markers provide indications on the strength of the synapse, the latter is a proxy for synaptic activity (Fig. 1a,b). Next, we chose calreticulin, ERGIC53, TGN38 and synaptopodin as markers for the ER, ERGIC, TGN and SA, respectively. Lastly, dendritic spines were identified by phalloidin labelling of actin. Four-colour images were acquired on proximal dendrites (within ~ 80 µm from the soma) of primary hippocampal neurons (17–20 days in vitro), co-labelled with a component of the secretory pathway, homer, either vGLUT1 or SYT1, and phalloidin. All channels, with the exception of phalloidin, were acquired in STED mode, to allow an accurate localisation of the structures within the confined post-synaptic space. Using this approach, we could identify all components of the secretory pathway in proximity to the post-synaptic site in the dendritic compartment, and observed that none was present at substantial levels in the pre-synaptic boutons (Fig. 1c,d, Supplementary Fig. 1). The brightness of their signals was significantly higher than the background due to non-specific binding of the secondary antibody (Supplementary Fig. 2). While the synaptopodin signal was clearly concentrated in the spine head, calreticulin, ERGIC53 and TGN38 appeared as sparse, but distinct, spots in both the dendritic shaft and spines. On average, the number of spots per spine head was three or four, with synaptopodin being the most abundant, while calreticulin and TGN38 were the least abundant (Supplementary Fig. 3).

### Post-synaptic structure correlates with the abundance of secretory pathway elements

**Figure 2:**
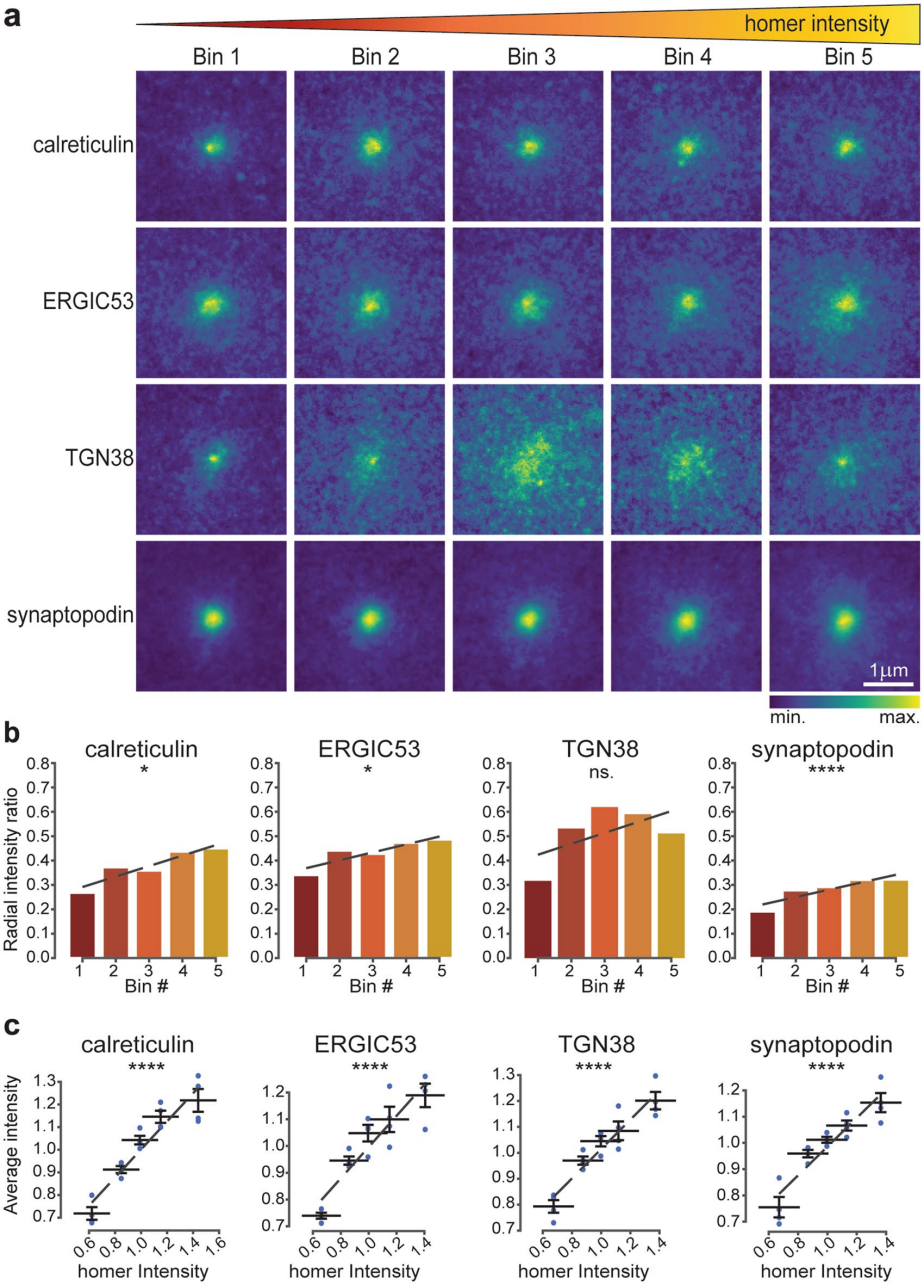
Correlation between post-synaptic strength and secretory pathway elements. (**a**) Average images for the different POIs based on five homer intensity bins displayed in 3 × 3 µm crops around the synaptic site. Bin 1 has the lowest homer intensity and bin 5 the highest. (**b**) Ratio between signal intensity at the periphery and at the centre of the average images, calculated by radial analysis. (**c**) Correlation of homer intensities and the average intensities of the POIs. Dashed lines are linear fits. All data were collected from four independent experiments. * = *p* < 0.05, ** = *p* < 0.01, *** = *p* < 0.001, **** = *p* < 0.0001, and ns. = non-significant. Whiskers represent S.E.M. Number of synapses per bin and Spearman’s rho are listed in Supplementary Table 1.

Having identified several components of the secretory pathway in proximity to post-synaptic sites, we set out to investigate whether the post-synaptic strength influences the distribution of the dendritic secretory machinery. To this aim, we used an approach already applied for similar tasks^50^ and described in Supplementary Fig. 4. Briefly, a 3 × 3 µm area centred on the synaptic sites, regardless of its position with respect to the post-synaptic compartment, was cropped, and the homer signal intensity within this crop was evaluated. The area dimension was chosen to include the whole post-synaptic site (considering an average spine length of ~ 1.5 µm) and to minimise the contribution from neighbouring synapses or structures. Crops were then sorted into five bins of equal size, based on homer signal intensity, and an average image of the protein of interest (POI) was generated for each bin. The average images represent the protein distribution around the synaptic sites, sorted by their respective homer intensity: a strong signal at the centre indicates a higher relative protein level in proximity of the synaptic site, while a more diffuse signal indicates that the protein is probably present in compartments more distant to the synaptic site, such as the dendritic shaft (Fig. 2a). Interestingly, while only a minor increase in the spread was noticed for calreticulin, ERGIC53 and synaptopodin at increasing homer intensities, TGN38 appeared vastly affected by low and intermediate post-synaptic strengths. To quantify this redistribution, a radial analysis was performed on the average images, and the ratio between the signal in the periphery and at the centre was calculated (Fig. 2b, Supplementary Fig. 5a). Low ratio values indicate a higher concentration of the POI at the synaptic site, while values close to 1 indicate a spread-out distribution. Such analyses revealed that calreticulin, ERGIC53, and synaptopodin exhibit a slight linear increase in their distribution in the area surrounding the synaptic site (the radial intensity profile ratio increased by 0.18, 0.15 and 0.13, respectively). On the other hand, TGN38 spread appeared to be modulated already by small increases in the post-synaptic strength (0.3 increase of radial intensity profile ratio between bin 1 and bin 3), but this modulation rapidly reached a plateau. From these results it could be concluded that the strength of the post-synapse has a minor influence on the distribution of the elements of the secretory pathway in the synaptic site.

To further investigate whether the observed effects on protein distribution are also connected to an increase in the protein abundance, the intensity of each POI within a bin was plotted against the intensity of the homer signal (Fig. 2c). A strong correlation was observed for all elements, suggesting that the abundance of these proteins is increased in the proximity of bigger post-synaptic sites. Altogether, these data indicate that homer intensity, as a proxy of the post-synaptic strength, differentially correlates with the distribution and abundance of elements of the secretory pathway.

### The abundance of post-synaptic secretory pathway elements also correlates with pre-synaptic strength

**Figure 3:**
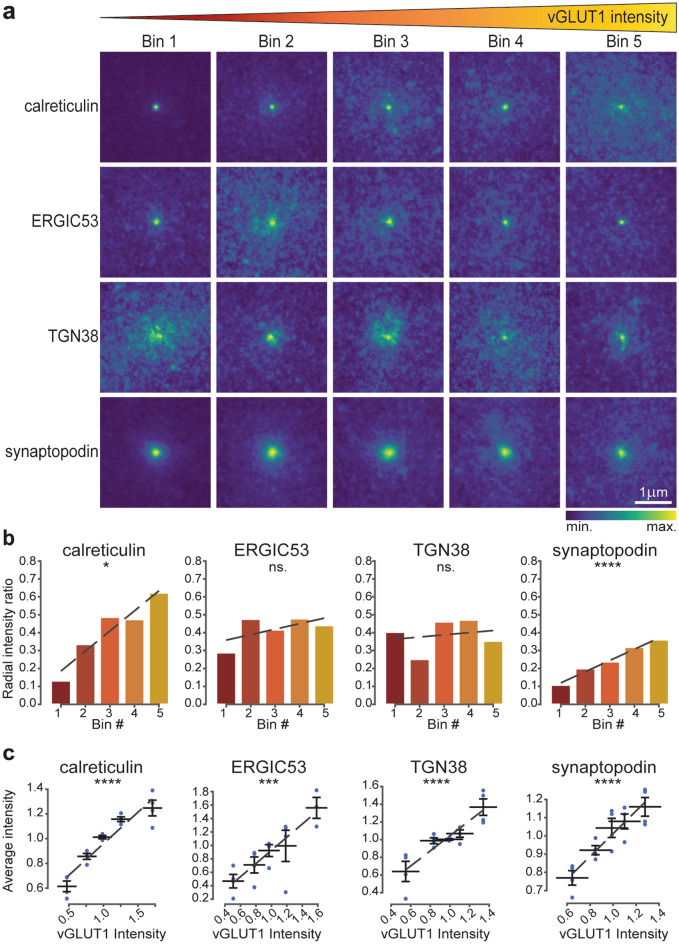
Correlation between pre-synaptic strength and secretory pathway elements. (**a**) Average images for the different POIs based on five vGLUT1 intensity bins displayed in 3 × 3 µm crops around the synaptic site. Bin 1 has the lowest vGLUT1 intensity and bin 5 the highest. (**b**) Ratio between signal intensity at the periphery and at the centre of the average images calculated based on radial analysis. (**c**) Correlation of vGLUT1 intensities and the average intensities of the POIs. Dashed lines are linear fits. All data were collected from four independent experiments. * = *p* < 0.05, ** = *p* < 0.01, *** = *p* < 0.001, **** = *p* < 0.0001, and ns. = non-significant. Whiskers represent S.E.M. Numbers of synapses per bin and Spearman’s rho are listed in Supplementary Table 1.

After having investigated the relationship between secretory pathway elements and post-synaptic site size, we wondered whether the same influence could be observed for the pre-synaptic site traits. The question is highly relevant, since in our experimental setup there is no significant correlation between pre- and post-synaptic site strength (Supplementary Fig. 6). Therefore, we performed the same analysis as conducted above, but we analysed the vGLUT1 intensity instead of that of homer, thereby estimating the size of the synaptic vesicle pool in the pre-synapse. Labelling of vGLUT1 was achieved using a primary nanobody, which provided a well-defined number of fluorophores per labelled structure^51^. The resulting averaged images for the different secretory elements showed a mixed pattern of responses (Fig. 3a,b, Supplementary Fig. 5b). Calreticulin and synaptopodin exhibit a linear redistribution at increasing vGLUT1 intensities, visible from both the average images and the radial analysis (the radial intensity profile ratios increased by 0.36 and 0.25, respectively). This increase was less pronounced, and was also non-linear, in the case of ERGIC53 (0.15 increase in radial intensity profile ratio), and was abolished for TGN38 (0.05 decrease in radial intensity profile ratio), whose distribution fluctuated between the different bins. Therefore, the influence of vGLUT1 abundance, as representation of pre-synaptic strength, on the distribution of post-synaptic elements of the secretory pathways appears heterogeneous.

When analysing the intensities of the POIs with respect to vGLUT1 intensity, all elements exhibited a clear linear correlation, which was particularly strong for calreticulin, TGN38, and synaptopodin (Fig. 3c). These data indicate that larger quantities of secretory pathway proteins are recruited into the post-synapses opposing strong pre-synapses. With the exception of TGN38, the higher quantity of POIs seems also to be reflected by their more dispersed distribution in the dendritic regions neighbouring the post-synaptic site.

### The distribution of post-synaptic secretory pathway elements is highly dependent on pre-synaptic activity

**Figure 4:**
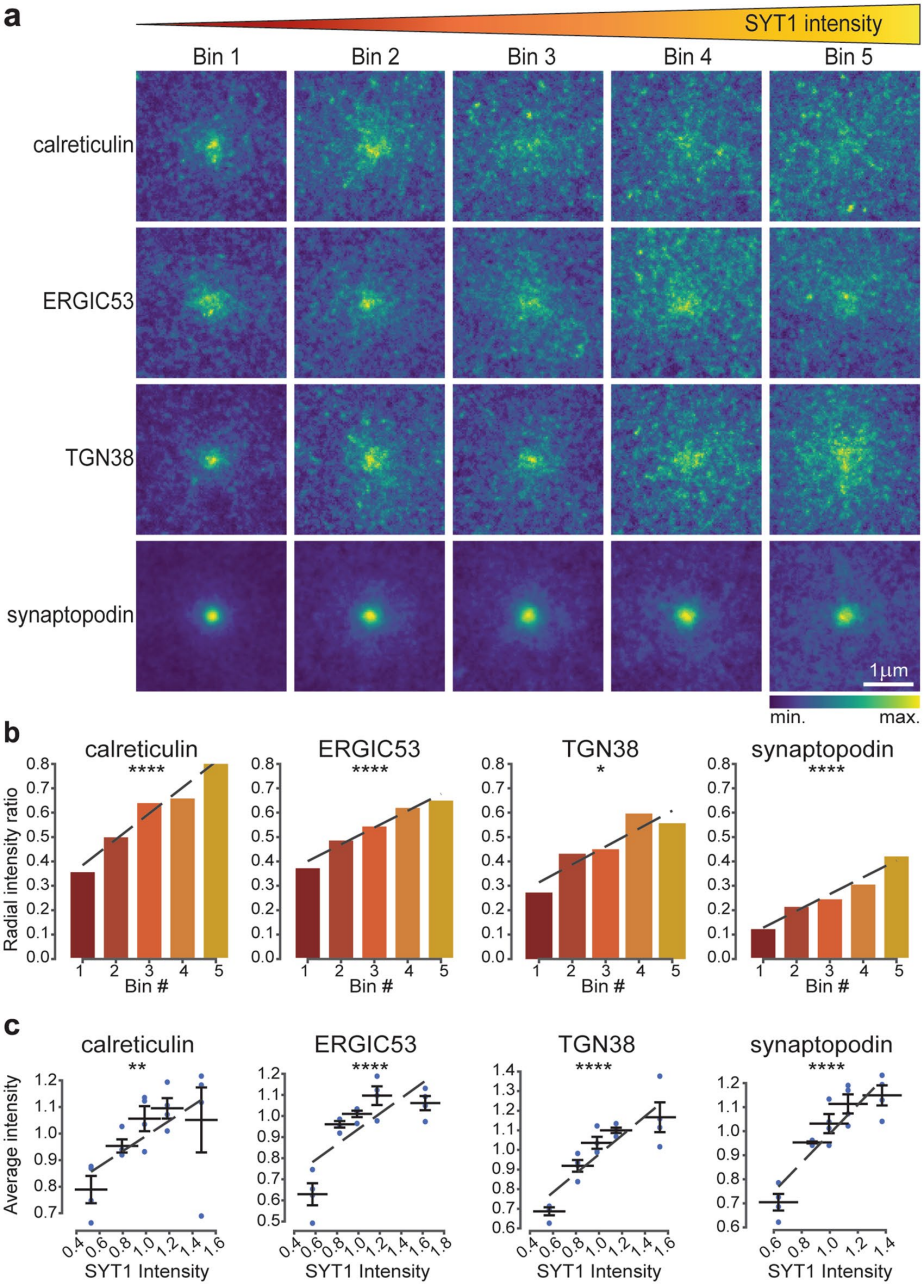
Correlation between pre-synaptic activity and secretory pathway elements. (**a**) Average images for the different POIs based on five SYT1 intensity bins displayed in 3 × 3 µm crops around the synaptic site. Bin 1 has the lowest SYT1 intensity and bin 5 the highest. (**b**) Ratio between signal intensity at the periphery and at the centre of the average images calculated based on radial analysis. (**c**) Correlation of SYT1 intensities and the average intensities of the POIs. Dashed lines are linear fits. All data were collected from four independent experiments. * = *p* < 0.05, ** = *p* < 0.01, *** = *p* < 0.001, **** = *p* < 0.0001, and ns. = non-significant. Whiskers represent S.E.M. Numbers of synapses per bin and Spearman’s rho are listed in Supplementary Table 1.

After having assessed the relationship between post- and pre-synaptic strength and secretory pathway components, we proceeded to address the existence of a functional correlation to synaptic activity. To this aim, we replaced vGLUT1 labelling by live-SYT1 staining in our experimental setup (Fig. 1b,d), which provided an estimate of synaptic activity based on the level of actively recycled vesicles. To our surprise, all POIs, including TGN38, showed a linear redistribution at increasing SYT1 intensities, as demonstrated by both average images and radial analysis (Fig. 4a,b, Supplementary Fig. 5c). For all POIs, the differences between the radial intensity profile ratios of bins 5 and 1 was higher than those observed for homer and vGLUT1 (Figs. 2b, 3b). This difference was least prominent for synaptopodin (0.3 increase in radial intensity ratio), and most prominent for calreticulin (0.45 increase in radial intensity ratio). Therefore, one can conclude that the localisation of all of the analysed post-synaptic elements of the secretory pathway is intimately linked to the amount of recycling vesicles and hence to pre-synaptic activity.

When correlating the POIs levels to SYT1 intensities, we found only a small correlation with calreticulin abundance in the region neighbouring the synaptic sites (Fig. 4c). On the contrary, the amounts of ERGIC53, TGN38, and synaptopodin were strongly correlated with SYT1 intensity. Taken together, these data indicate that the secretory organelles at the synapse are increasingly dispersed with increasing amounts of recycling vesicles, though the effect of activity on their abundance varies depending on the organelle.

### Post-synaptic strength strongly modulates the secretory machinery in mushroom spines

**Figure 5:**
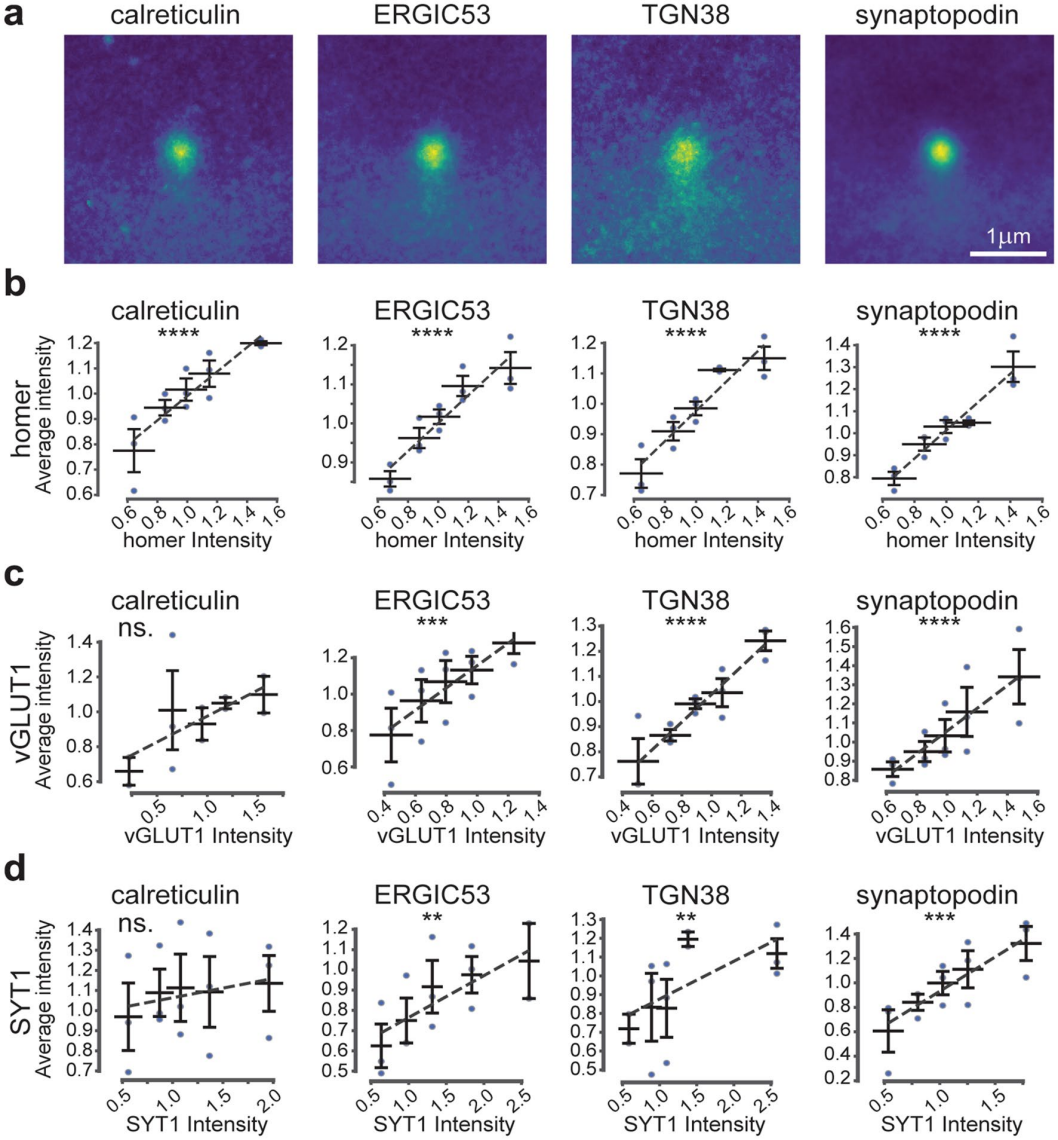
Correlations of secretory elements in mushroom spines. (**a**) Average 3 × 3 µm images of POIs in mushroom spines as defined by manual segmentation. Images were vertically aligned with the spine head at the centre and the dendritic shaft at the bottom. All data were collected from three independent experiments. (**b**–**d**) Average intensity correlations after manual spine segmentation and classification as mushroom type spines. Correlation of the average intensities of calreticulin, ERGIC53, TGN38, and synaptopodin with average intensities of homer (**b**), vGLUT1 (**c**), and SYT1 (**d**). Where * = *p* < 0.05, ** = *p* < 0.01, *** = *p* < 0.001, **** = *p* < 0.0001, and ns. = non-significant. Whiskers represent S.E.M. Number of synapses per bin and Spearman’s rho are listed in Supplementary Table 1.

The analyses conducted so far assessed how synaptic strength and activity relate to the abundance of secretory pathway elements in the synapse and the extent of their dispersion, without considering the actual size of the spines containing them. Furthermore, it did not discriminate between different spine morphologies. To account for this, a manual classification and segmentation of spines was performed based on the actin (phalloidin) staining. In this way, spines that had a clear mushroom or stubby morphology were selected from the 3 × 3 µm crops, and their axes were aligned. The corresponding average images confirmed the results obtained with the automatic analysis in the previous figures, and demonstrated that the signal of the POIs derives predominantly from the dendritic, rather than the axonal compartment (Fig. 5a and Supplementary Fig. 7). The POIs were enriched in proximity to the synaptic site, with TGN38 having the most dispersed distribution, and synaptopodin the most confined distribution in both spine types. Furthermore, the average intensity for most of the POIs was comparable between both mushroom and stubby spines, and only synaptopodin showed a slight enrichment in mushroom spines, as expected from the literature (Supplementary Fig. 8)^17,20^.

The analysis of the manually segmented images indicated that the levels of all POIs strongly correlated with homer levels, regardless of the spine type. Moreover, in mushroom spines most POIs correlated with vGLUT1 intensity (Fig. 5b,c and Supplementary Fig. 9). This positive correlation is most likely due to the fact that the number of POI spots in the spine head grows with the size of the post-synaptic site. However, the size of the spots decreased with increasing post-synaptic density size (Supplementary Fig. 10), suggesting the presence of more but smaller organelles. These results confirmed the previously observed correlations between the abundance of secretory pathway elements and markers of pre- and post-synaptic strength, as was assessed with the automatic analysis presented above (Figs. 2, 3).

When analysing the synaptic activity in the manually segmented images, however, a correlation was observed exclusively in mushroom spines, and only for ERGIC53, TGN38, and synaptopodin (Fig. 5d). The previously observed correlation for calreticulin was lost in both spine types. Therefore, these results suggest that the structure of the pre- and post-synaptic site has a high impact in regulating the amount of secretory pathway elements present in the neighbouring post-synaptic regions, and that pre-synaptic activity has differential effects, depending on the post-synaptic morphology.

## Discussion

Neurons have a highly elaborated network of secretory elements that allows for locally-independent production and processing of proteins^5,8,10,52,53^. However, the extent to which synaptic activity, size and morphology steer local protein synthesis machinery is not fully understood^11,54^. Here we systematically analysed the abundance and distribution of several secretory elements at synaptic sites by means of STED optical nanoscopy, in dependence of proxies of synaptic strength and activity. With quantitative image analysis, we investigated the correlations between the abundance and distribution of calreticulin (endoplasmic reticulum marker), ERGIC53 (ER-Golgi intermediate compartment marker), TGN38 (trans-Golgi network marker), and synaptopodin (spine apparatus marker), and the pre-synaptic markers vGLUT1 or SYT1, as well as the post-synaptic marker homer.

The use of optical nanoscopy has the advantage of providing molecular specificity and allowing the unambiguous discrimination of organelles, which can be difficult in electron microscopy^27^. Furthermore, a higher throughput required for extensive analyses, and the visualisation of the entire three-dimensional synaptic site are both more easily achievable with optical microscopy. However, in case of overlapping structure, signal coming from superimposed compartments or synaptic sites could be misinterpreted, since the axial resolution in two-dimensional STED is limited to 500–700 nm. Moreover, the results are influenced by the specificity of the markers for the structures of interest and by the precision of the related antibodies, which were validated by western blot and STED imaging in cell lines (Supplementary Fig. 11). In the context of this study, this is relevant for synaptopodin, since it is not uniquely a spine apparatus marker, and it can be found in spines prior to the formation of the spine apparatus, as well as in the axon initial segment^15,55^. Lastly, the presence of non-specific binding of the primary antibody cannot be completely ruled out and could only be verified on cultures derived from knock-out animals.

Our data analysis was largely automated, thus limiting biases in the image quantification and evaluation. With this approach, thousands of synaptic sites could be analysed to gain information on the behaviour of the “average” synaptic site. However, rare events are not represented, as they would require the manual inspection of each individual site in order to be identified. One bias of our analysis is that larger post-synaptic sites, particularly when considering the manually selected mushroom or stubby spines, will intrinsically lead to a higher occupancy of the image space and thus to higher protein levels (assuming that the proteins are homogeneously distributed in the spine head). However, none of the proteins we analysed exhibits a volume labelling-like staining, limiting this drawback. The manual identification of post-synaptic sites might also intrinsically predispose to the selection of bigger synaptic sites, whose behaviour may not be representative of all synapses. This influence might explain, to some extent, the differences observed between automated and manually selected synapse. Lastly, it should be mentioned that in our experimental setup no correlation between the levels of vGLUT1 and homer was observed (Supplementary Fig. 6). Therefore, the responses of the POIs to pre- and post- synaptic strength can be considered independently from each other.

STED imaging showed a higher concentration of some of the chosen markers in the soma or along the apical dendrite. Nevertheless, in our experimental setup all POIs could be identified unambiguously in the dendritic shafts and at post-synaptic compartments. According to previous studies, the ERGIC was proposed to be rarely present in dendritic spines^26^. However, it should be noted that in electron microscopy the ERGIC resembles endosomes, which are often found in spines, and therefore this similarity might lead to misinterpretation of the images^27^. Furthermore, fine ERGIC53 puncta could be easily overseen or mislocalised when using diffraction-limited light microscopy. Regarding TGN38, a detailed description of its presence at synaptic sites has so far been reported only in overexpression experiments, and was not investigated in detail at endogenous levels^2,33,35^.

**Figure 6:**
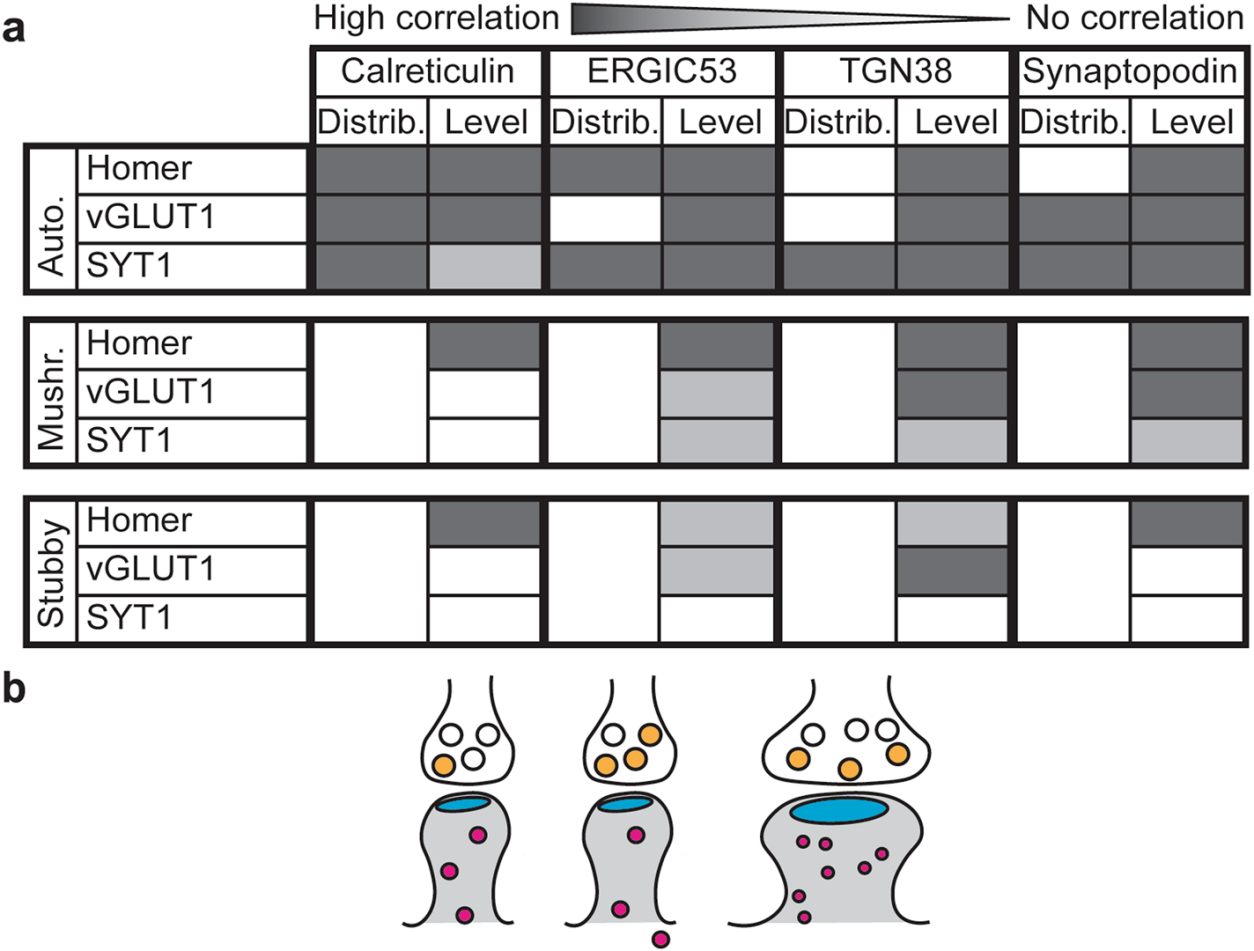
Summary of effects and proposed model. (**a**) Summary of the correlation between the elements of the secretory pathway (in terms of distribution and levels), and post-synaptic strength (homer), pre-synaptic strength (vGLUT1), or pre-synaptic activity (SYT1) for the different analysis performed (automatic analysis of all synapses, or manual analyses of mushroom spines and stubby spines). The different shades of grey correspond to high correlation/influence (darker grey), or less (light grey) to no (white) correlation/influence. (**b**) Model for the effect of synaptic strength and activity on the elements of the secretory pathway. Increased activity initially mobilizes the locally available resources, whose availability increases to support spine enlargement and maintenance.

Overall, we found strong correlations of the expression levels of the components of the secretory pathway to synaptic strength and activity, and in particular to the post-synaptic size (Fig. 6a). However, several details should be discussed for each of the individual markers. Calreticulin distribution was dependent on post-synaptic size, and even stronger effects were observed for pre-synaptic strength and activity. The amounts of the protein were dependent on the strength of the synapse, but correlated less with synaptic activity. The correlation with pre-synaptic markers was heavily abolished when analysing mushroom and stubby spines specifically. These effects are in line with a model in which synaptic activity recruits the local ER resources to the synaptic site, thereby explaining the correlation between activity and calreticulin distribution. Later on, persistent activity results in the recruitment of the ER from more distant areas, thereby leading to an increase in ER abundance and explaining the correlation between calreticulin levels and synaptic strength. This model is in agreement with the evidence that the ER has a high turnover and that it primes new spines for future plasticity, contributing to LTP^14,15,56^.

ERGIC53 levels directly correlated with the levels of all three synaptic markers, also in mushroom spines, and its distribution was mainly affected by pre-synaptic activity. These results suggest that ERGIC-mediated protein trafficking is required for the maintenance of synaptic strength (and presumably for plasticity), and that it responds readily to synaptic activity, in agreement with previous investigations^29^.

The TGN38 behaviour was to some extent similar to that of ERGIC53. The TGN38 distribution in connection to post-synaptic strength appears highly sensitive to changes for low homer intensities, and becomes saturated for high values. The pre-synaptic strength appears to have no effect on the TGN38 localisation. Pre-synaptic activity correlated strongly with both TGN38 distribution and levels, but this correlation was strongly reduced when manually segmenting spines. This difference might be ascribed to the analysis itself: (1) mushroom and stubby spines that can be identified unambiguously may already be relatively mature, and therefore maintenance mechanisms will predominate over plasticity; (2) both spine types generally have the synaptic contact in the head region, and hence to some distance from the dendritic shaft, whose contribution in the analysis is therefore reduced. Therefore, a potential recruitment of TGN38 in the shaft region would be quantified in the automatic analysis, but would not be measured in the analysis of the individual spine types. We therefore propose that TGN38 is mobilised in the proximity of the post-synapse, in relation to activity, but not necessarily in the spine head. Some of the TGN38 molecules may have been synthesised in response to activity^36^, albeit this is only speculative, since the age of the compartments was not analysed in our experiments.

In principle, one would expect a strong similarity between the ERGIC and TGN compartments. However, neurons also have unconventional secretion pathways that bypass the TGN^57^, thereby explaining why these compartments do not behave in an identical fashion.

Synaptopodin showed a strong correlation to all synaptic markers and in both automatically and manually selected mushroom spines. On the other hand, synaptopodin distribution seemed to be less influenced by post-synaptic strength but more by pre-synaptic modulations. Because of its functions, synaptopodin is specifically accumulated in the spine head and neck in mushroom spines^17,18,20^. Therefore, it is not surprising that all tested synaptic characteristic proxies showed a good correlation with synaptopodin abundance, and that even at increasing post-synaptic strength, synaptopodin stayed in close synaptic proximity. However, it is important to consider that the presence of synaptopodin in the spines is not necessarily related to the presence of the SA, and therefore the results might be partially connected to the ability of synaptopodin to bind the actin cytoskeleton^17–20^.

Altogether, our data suggest that the pre-synaptic activity has the highest influence on the distribution of secretory pathway elements in the proximity of post-synaptic sites, while the post-synaptic architecture has the highest influence on their abundance. Therefore, we propose a model in which increased synaptic activity initially mobilises the locally available resources to the synaptic area. Sustained activity then leads to increases in synapse strength, and promotes the recruitment of more secretory organelles that are important for the maintenance of the large synapses (Fig. 6b). However, it should be noted that the observed effects are not homogenous for all the components of the secretory pathway, further proving the presence of unconventional pathways in neurons^57^.

A basic conclusion of our study is that both pre- and post-synaptic features correlate in multiple ways to the post-synaptic secretory pathway. Further work will be required to understand the exact synaptic mechanisms modulating protein secretion in the context of synaptic plasticity and activity. Finally, the behaviour of other organelles, such as the Golgi outposts and the recycling endosomes, should also be considered in the future.

## Methods

### Sample preparation

Cultures of dissociated rat hippocampal primary neurons were prepared from postnatal P0–P2 Wistar rats of either sex and as described in^58^. Procedures were performed in accordance with the Animal Welfare Act of the Federal Republic of Germany (Tierschutzgesetz der Bundesrepublik Deutschland, TierSchG) and the Animal Welfare Laboratory Animal Regulations (Tierschutzversuchsverordnung). According to the TierSchG and the Tierschutzversuchsverordnung no ethical approval from the ethics committee is required for the procedure of sacrificing rodents for subsequent extraction of tissues, as performed in this study. The procedure for sacrificing P0–P2 rats performed in this study was supervised by animal welfare officers of the Max Planck Institute for Medical Research (MPImF) and conducted and documented according to the guidelines of the TierSchG (permit number assigned by the MPImF: MPI/T-35/18).

For the labelling of the actively recycled vesicle pool, mature cultures (17–20 days in vitro) were incubated for 1 h with a Atto647N-labelled mouse antibody against the luminal domain of synaptotagmin 1 (Synaptic Systems, cat. 105 311AT1, 1:500 in culture medium). Afterwards cultures were washed three times in warm ACSF (126 mM NaCl, 2.5 mM KCl, 2.5 mMCaCl_2_, 1.3 mM MgCl_2_, with 30 mM Glucose, 27 mM Hepes). Labelled and unlabelled samples were fixed for 30 min in 4% PFA in PBS, pH 7.4, and quenched for 5 min in quenching buffer (PBS, 100 mM glycine, 100 mM ammonium chloride). Cells were permeabilised for 5 min in 0.1% Triton X-100 and blocked with 1% BSA for 1 h. Samples were incubated with primary antibody dilutions in PBS for 1 h at room temperature. Primary antibodies used were: homer1 (Synaptic Systems, cat. 160 004, 1:500 dilution), calreticulin (Cell Signaling, cat. 12238S, 1:100 dilution), ERGIC-53/p58 (Sigma E1031, 1:200 dilution), TGN38 (Sigma, cat. T9826, 1:100 dilution), synaptopodin (Synaptic Systems, cat. 163,002, 1:200 dilution). Samples were washed and incubated with secondary antibody dilutions (Alexa Fluor 488 anti-guinea pig, Thermo Fisher, cat. A-11073 and Alexa Fluor 594 anti-rabbit, Thermo Fisher, cat. A-21207), Phalloidin (Alexa Fluor 405 Thermo Fisher, cat. A30104; all at 1:100 dilution) and single domain antibody against vGLUT1 (Synaptic Systems, cat. N1602-Ab635P-S, 1:200 dilution), for 1 h at room temperature. After washing, samples were embedded in Mowiol supplemented with DABCO.

### Imaging

Samples were imaged on an Abberior expert line (Abberior Instruments GmbH, Germany) with pulsed STED lines at 775 nm and 595 nm, excitation lasers at 355 nm, 405 nm, 485 nm, 580 nm, and 640 nm, and spectral detection. Detection windows were set to 650–725 nm, 600–630 nm, 505–540 nm, and 420–475 nm to detect Atto647N, Alexa Fluor 594, Alexa Fluor 488 and Alexa Fluor 405, respectively. Images were acquired with a 100x/1.4 NA magnification oil immersion lens. Pixel size was set to 30 nm, pinhole to 100 µm (1 AU). Laser powers, line accumulations and dwell times were kept consistent throughout the entire study. Alexa Fluor 594 and Atto647N were imaged semi-simultaneously during a first acquisition with STED at 775 nm, while Alexa Fluor 488 was imaged afterwards using STED at 595 nm. Confocal acquisition of the Alexa Fluor 488 channel was performed in all the image sequences to monitor lateral drift. Axial drift was minimized by the Z-focus drift compensation unit.

### Image processing

Images were visualised and processed with Imspector (Abberior Instruments GmbH, Göttingen Germany) and ImageJ 1.52p (imagej.nih.gov/ij/). In the figures, images are shown as smoothed data with a low pass Gaussian filter and 5% background subtraction. Brightness was adjusted uniformly throughout the images. For analysis of the radial intensity in average images, the ImageJ plugin radial profile plot was used. The ratio was calculated by dividing the average intensity of peripheral 35–45 radial pixels by the average intensity of the centre 10 pixels.

### Data analysis

Analyses were performed in Matlab (MathWorks, Natick, MA, USA) and Python (Python Software Foundation) (Supplementary Fig. 4). For the automatic spine analysis, the homer, vGLUT1 or SYT1 channels were manually thresholded and used to locate the coordinates of the synapses within the image. Square regions of 3 × 3 µm, centered on the homer, vGLUT1 or SYT1 puncta were excised in order to obtain the average images shown in Figs. 2, 3, 4. The radial intensity values were then measured in the average images, and a ratio was calculated between a 10 pixel-wide ring in the periphery and in the center of the image. The individual image segments were binned in five ordinal groups based on the mean fluorescence intensity of the pre- or post-synapse markers, to include a similar number of synapses. For each experiment, the mean fluorescence was calculated in each bin, and was normalized to the median intensity of the experiment.

For the manual segmentation of spines (Fig. 5 and Supplementary Fig. 7), spines were selected based on the homer signal, which indicates the post-synaptic site, and on the actin signal, which provides the overall organization of the spine and of the dendritic shaft. A smaller selection area, of 3 × 3 µm, centred on the homer puncta, was then processed further. The identity of the synapse was assigned (mushroom or stubby), and then the main landmarks of the spine were marked manually. These included the top, bottom, right and left borders of the head of the spine, the top and bottom points of the neck (in mushroom spines), as well as the position of the junction between the shaft and the spine neck. The spines were then aligned, relying on these landmarks, and they were overlaid, resulting in the images shown in Fig. 5 and Supplementary Fig. 7. The fluorescence intensity for the POIs and the synaptic markers was calculated as above. For the calculation of protein spot size, the images were automatically segmented into spots using a wavelet transformation with a Spot Detection plugin for icy^59,60^. The scale 2 and an 80% threshold were used. All images were thresholded to remove background signal; regions above an empirically defined threshold were treated to contain real signals for further analysis. Outliers exceeding the range of mean ± 3 standard deviations were excluded from the analyses.

## Supplementary information

Supplementary Information 1.
