# Supplementary material for: Synaptic activity and strength are reflected by changes in the post-synaptic secretory pathway
Source: Sci Rep. 2020 Nov 25;10:20576. doi: 10.1038/s41598-020-77260-2 (PMC7688657; doi:10.1038/s41598-020-77260-2)
Supplement: Supplementary file 1 — Supplementary Information 1. [file 41598_2020_77260_MOESM1_ESM.pdf]

Supplemental Information:

Synaptic activity and strength are reflected by changes in the post-synaptic secretory pathway

Clara-Marie Gürth [1, 2, #], Tal M. Dankovich [3, #], Silvio O. Rizzoli [3], Elisa D'Este [4, *]

[1] Department of NanoBiophotonics, Max Planck Institute for Biophysical Chemistry, Am Fassberg 11, 37077 Göttingen, Germany

[2] Department of Optical Nanoscopy, Max Planck Institute for Medical Research, Jahnstr. 29, 69120 Heidelberg, Germany

[3] Institute for Neuro- and Sensory Physiology, University Medical Center Göttingen, Humboldtallee 23, 37073 Göttingen, Germany

[4] Optical Microscopy Facility, Max Planck Institute for Medical Research, Jahnstr. 29, 69120 Heidelberg, Germany

[#] Contributed equally to this work

* Corresponding author: elisa.deste@mr.mpg.de

Supplementary Table 1: List of statistical values and number of synapses per individual experiments.

Type of analysis	**Synaptic marker**	**POI**	**Synapses /bin**	**Total synapses**	**Rho**	**P (spearmen)**
**Automatic analysis of all synapses**	homer	calreticulin	643	3215	0.956	0.00000000
		ERGIC53	680	3400	0.889	0.00000016
		TGN38	379-380	1899	0.925	0.00000000
		synaptopodin	542-543	2712	0.968	0.00000000
		vGLUT1	1138	5690	0.435	0.05505578
		SYT1	1156-1157	5781	0.754	0.00012247
	vGLUT1	calreticulin	487-488	2439	0.956	0.00000000
		ERGIC53	265-266	1326	0.765	0.00013235
		TGN38	117-118	588	0.876	0.00000039
		synaptopodin	159-160	796	0.833	0.00000492
	SYT1	calreticulin	249	1245	0.656	0.00168277
		ERGIC53	176-177	883	0.784	0.00004172
		TGN38	152-153	763	0.895	0.00000009
		synaptopodin	213-214	1068	0.882	0.00000025
**Analysis of mushroom spines**	homer	calreticulin	186	930	0.820	0.0000034
		ERGIC53	208	1040	0.901	0.0000001
		TGN38	122	610	0.860	0.0000006
		synaptopodin	140	700	0.840	0.0000016
	vGLUT1	calreticulin	99	495	0.351	0.0546565
		ERGIC53	100	500	0.583	0.0009192
		TGN38	75	375	0.781	0.0000124
		synaptopodin	79	395	0.705	0.0000665
	SYT1	calreticulin	87	435	0.026	0.5600091
		ERGIC53	107	535	0.448	0.0087806
		TGN38	47	235	0.482	0.0083918
		synaptopodin	61	305	0.687	0.0001321
	head size	calreticulin size	182-183	914	0.550	0.001554104
		ERGIC53 size	209-210	1047	0.761	0.00002167
		TGN38 size	131-132	657	0.001	0.90780719
		synaptopodin size	139-140	696	0.820	0.00000339
		calreticulin number	182-183	914	0.705	0.000008845
		ERGIC53 number	209-210	1047	0.901	0.00000006
		TGN38 number	131-132	657	0.653	0.0002669
		synaptopodin number	139-140	696	0.964	0.0000000
**Analysis of stubby spines**	homer	calreticulin	61	305	0.820	0.0000034
		ERGIC53	81	405	0.651	0.0002730
		TGN38	51	255	0.502	0.0030688
		synaptopodin	53	265	0.860	0.0000006
	vGLUT1	calreticulin	24	120	0.193	0.1757708
		ERGIC53	46	230	0.687	0.0001321
		TGN38	36	180	0.820	0.0000034
		synaptopodin	31	155	0.260	0.0620052
	SYT1	calreticulin	37	185	0.129	0.1874141
		ERGIC53	34	170	0.316	0.0363651
		TGN38	15	75	0.396	0.0378569
		synaptopodin	22	110	0.193	0.1325609

Supplementary Table 1 continued: List of statistical values and number of synapses per individual experiments.

Type of analysis	**Synaptic marker**	**POI**	**Synapses /bin**	**Total synapses**	**Rho**	**P (spearmen)**
**Automatic analysis of all synapses - Radial redistribution**	homer	calreticulin	643	3215	0.899	0.03738607
		ERGIC53	680	3400	0.899	0.03738607
		TGN38	379-380	1899	0.300	0.62383766
		synaptopodin	542-543	2712	0.999	0.00000000
	vGLUT1	calreticulin	487-488	2439	0.899	0.03738607
		ERGIC53	265-266	1326	0.499	0.39100221
		TGN38	117-118	588	0.199	0.74706007
		synaptopodin	159-160	796	0.999	0.00000000
	SYT1	calreticulin	249	1245	0.999	0.00000000
		ERGIC53	176-177	883	0.999	0.00000000
		TGN38	152-153	763	0.899	0.03738607
		synaptopodin	213-214	1068	0.999	0.00000000

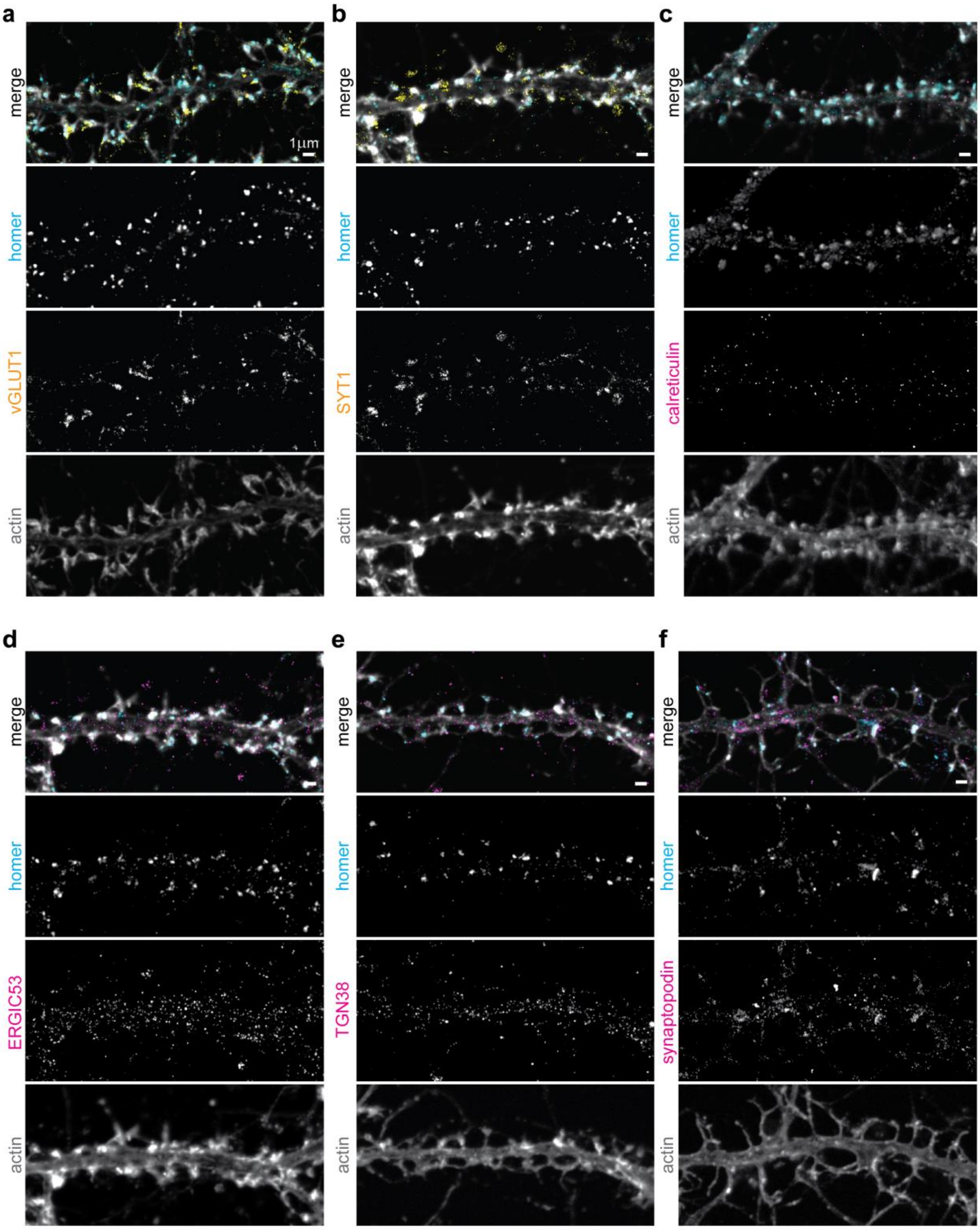

Supplementary Figure 1: Representative images of vGLUT1 and SYT1 compared to homer, and of the POIs stainings. Representative STED images of dendrites in mature hippocampal neuronal cultures. Samples were all stained with homer, phalloidin (actin, confocal), with pre- and post-synaptic sites markers vGLUT1 **(a)** or SYT1 **(b)**, and for the proteins of interest: calreticulin **(c)**, ERGIC53 (same dendrite as shown in b) **(d)**, TGN38 **(e)**, and synaptopodin **(f)**. Scale bars: 1 µm.

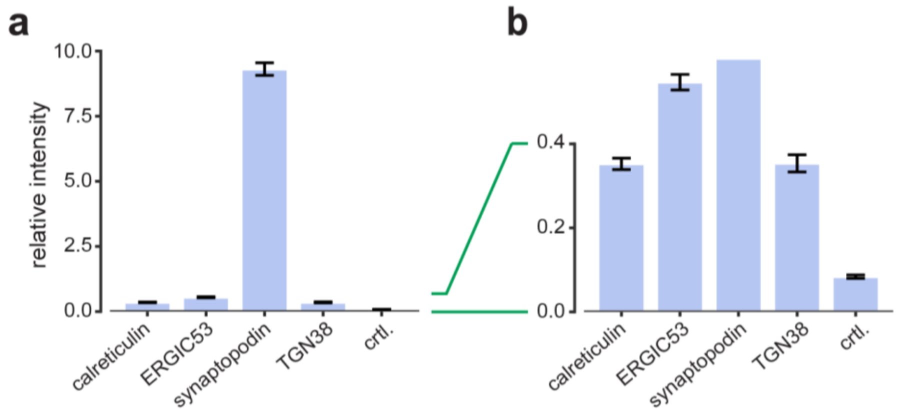

Supplementary Figure 2: Comparison of average intensities of the individual POIs. Average staining intensity of the different POIs and control samples in which the primary antibody was omitted. Comparison of all stainings (**a**) and close up on the lower intensities (**b**). All data was collected from 3 independent experiments. Whiskers represent S.E.M.

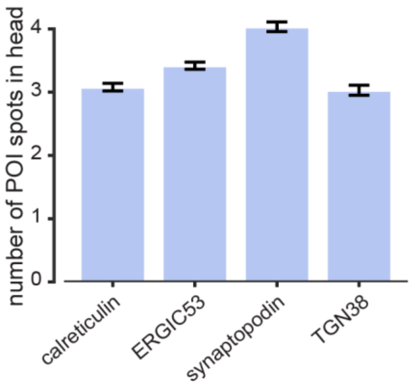

Supplementary Figure 3: Average spot number of secretory elements in mushroom spines. Average numbers of spots of calreticulin, ERGIC53, TGN38, and synaptopodin inside the spine head after manual segmentation. All data was collected from 3 independent experiments. Whiskers represent S.E.M.

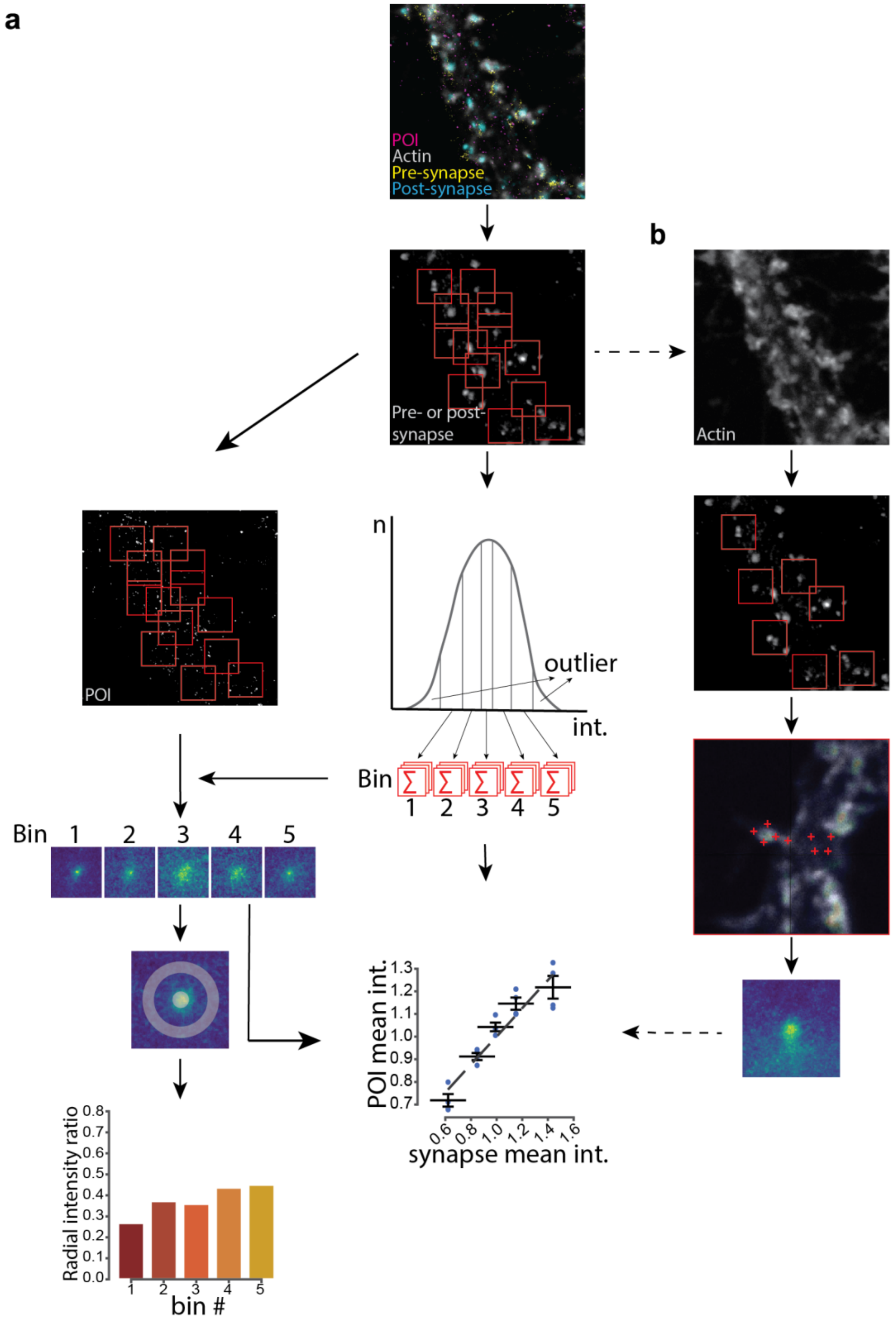

Supplementary Figure 4: Image analysis workflow. 4-colour images were acquired of the protein of interest (magenta), actin (white) and pre- and post-synaptic markers (yellow and cyan respectively). (a) Automatic analysis: the pre-/post-synaptic markers were used to identify synaptic puncta in the images. 3 x 3 µm square regions were extracted for each synapse and pooled into five bins based on increasing fluorescence intensity of the synaptic

markers. The POI images were averaged for each bin and correlated to the average intensity of the synaptic markers. Alternatively, a ratio was calculated between the outer rim and the central area of each average image to determine the tendency of the POI to localize to the synapse proximity. (b) Manual analysis: the post-synaptic marker was used to locate dendritic spines and extract 3 x 3 µm square regions as in (a). The actin channel of the images was then used to manually determine the borders of the spines in order to align them to a similar orientation before subsequent analysis.

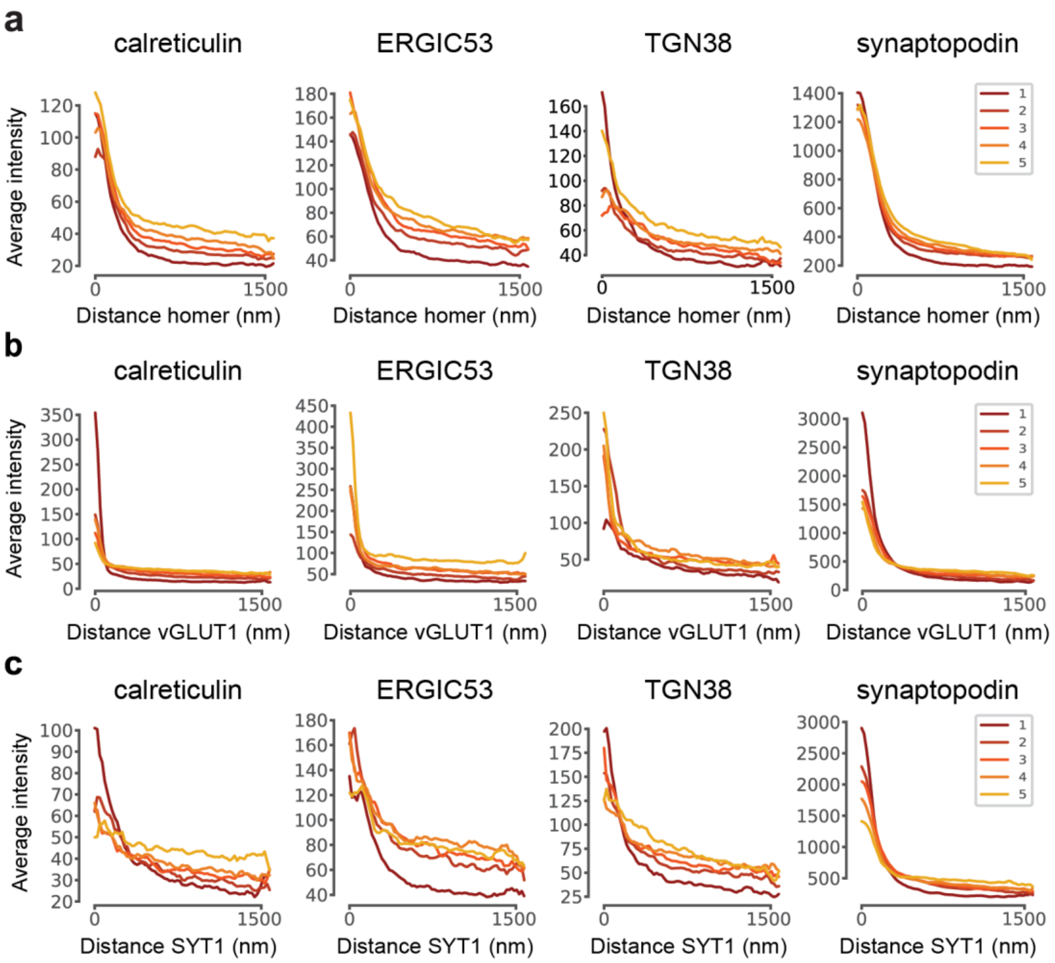

Supplementary Figure 5: Radial profile plots of the average intensity images. Radial profiles for bins of each POI: homer (**a**), vGLUT1 (**b**), and SYT1 (**c**). Bins 1-5 (dark red-yellow) were used for calculating the ratios depicted in Fig. 2b, 3b, and 4b, respectively.

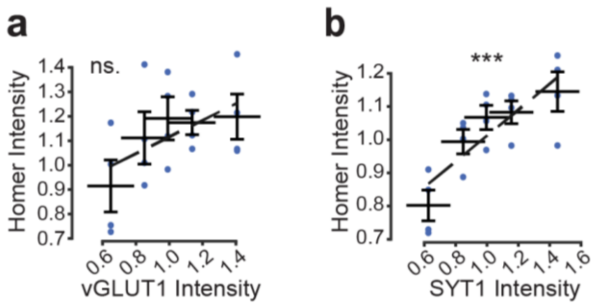

Supplementary Figure 6: Intensity correlations of pre-and post-synaptic markers. Correlation of homer intensities and the average intensities of vGLUT1 (**a**) and SYT1 (**b**). Dashed lines are the linear fits. All data was collected from four independent experiments. Where *=p<0.05, **=p<0.01, ***=p<0.001, ****=p<0.0001, and ns.=non-significant. Whiskers represent S.E.M. Numbers of synapses per each bin and Spearman's rho are listed in Supplementary Table 1.

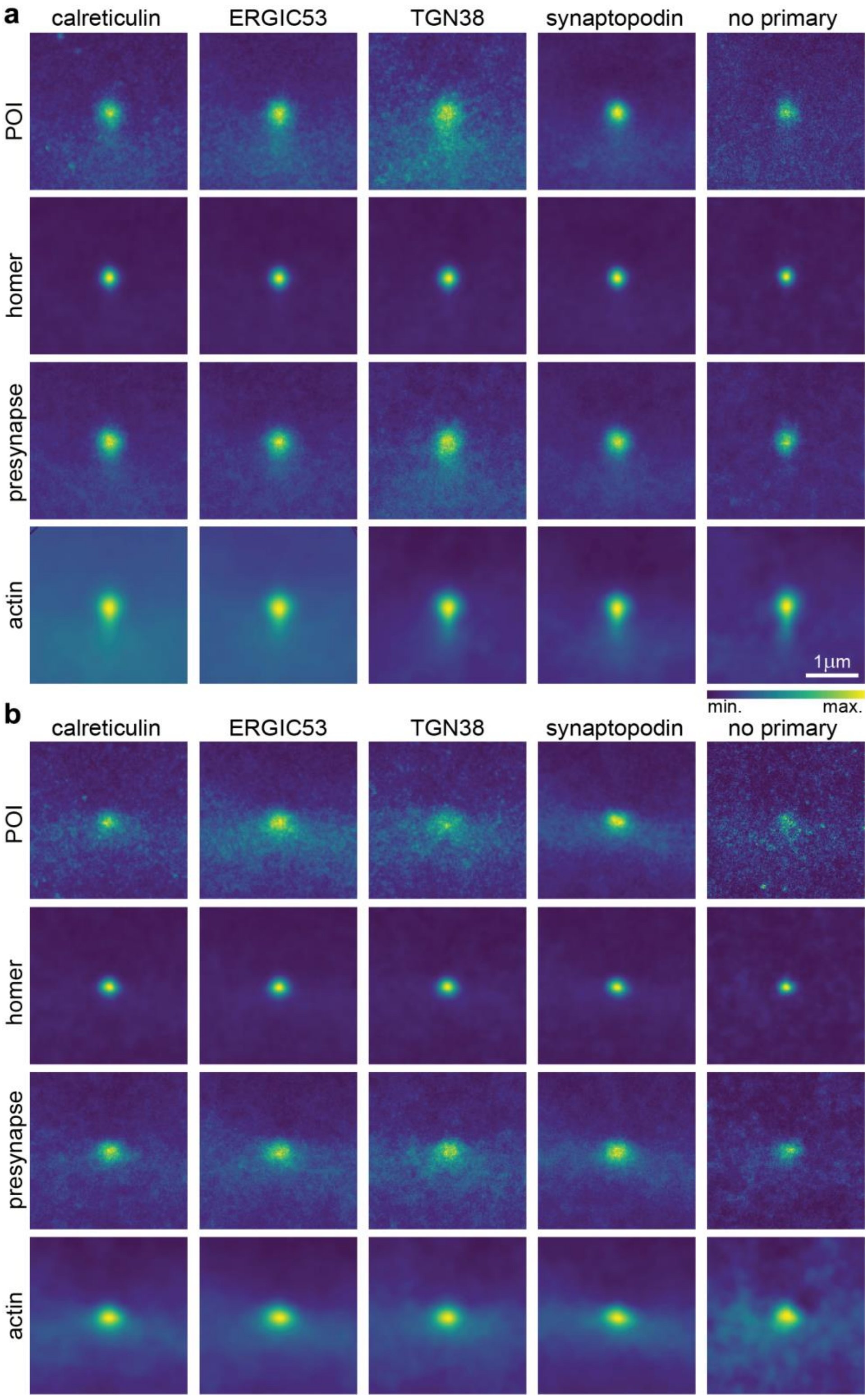

Supplementary Figure 7: Average images of spines after manual segmentation. Average images of mushroom **(a)** and stubby **(b)** spines of the POIs, homer, average pre-synaptic markers vGLUT1 and SYT1, and phalloidin. Intensities have been adjusted for each image. Average images of the POIs shown in panel (a) are the same average images depicted in Fig. 5a.

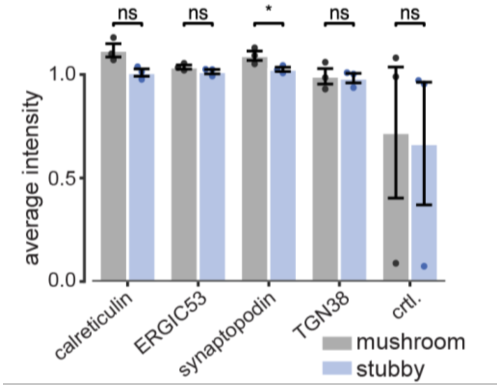

Supplementary Figure 8: Comparison of average intensities of mushroom and stubby spines. Average staining intesities of POI normalised to experimental means. Whiskers represent S.E.M.

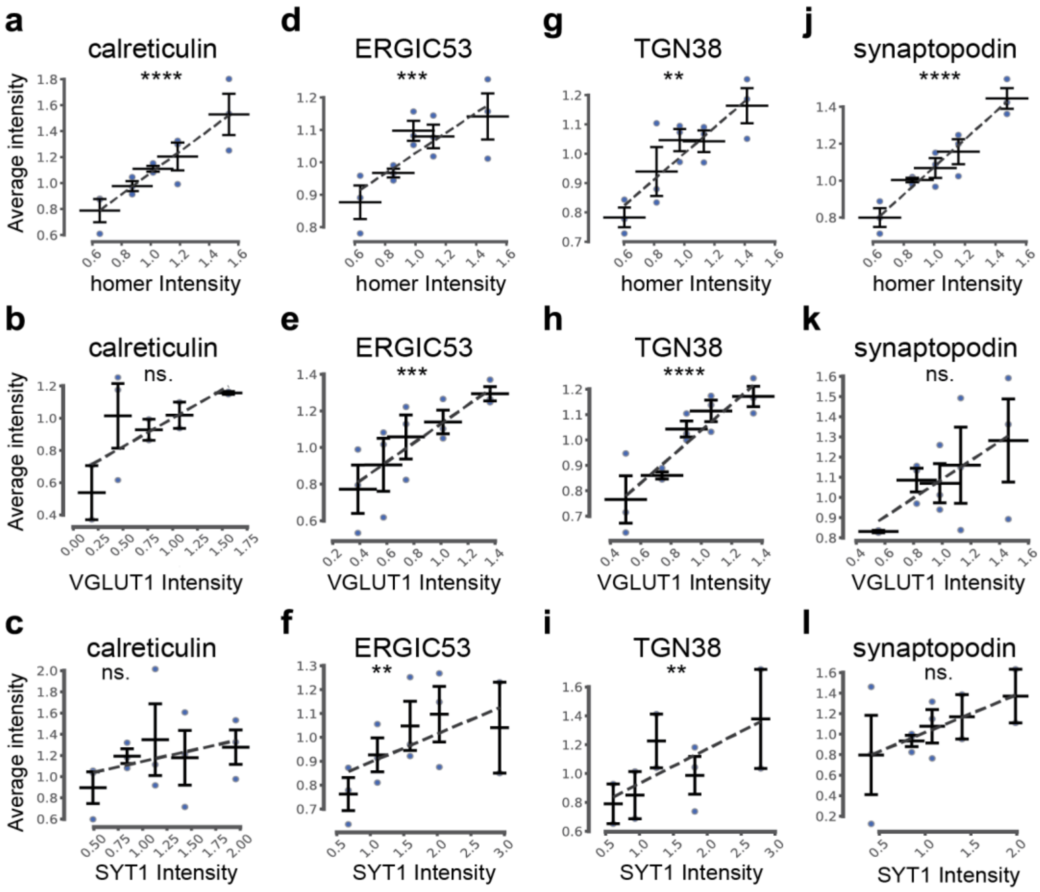

Supplementary Figure 9: Correlations of secretory elements at stubby spines. (a-l) Average intensity correlations after manual spine segmentation and classification of stubby type spines. Average intensities of calreticulin (a-c), ERGIC53 (d-f), TGN38 (g-i), and synaptopodin (j-l) correlated with average intensities of homer (a,d,g,j), vGLUT1 (d,e,h,k), and SYT1 (c,f,i,l). Dashed lines are linear fits. All data were collected from three independent experiments. Where *=p<0.05, **=p<0.01, ***=p<0.001, ****=p<0.0001, and ns.=non-significant. Whiskers represent S.E.M. Numbers of synapses per each bin and Spearman's rho are listed in Supplementary Table 1.

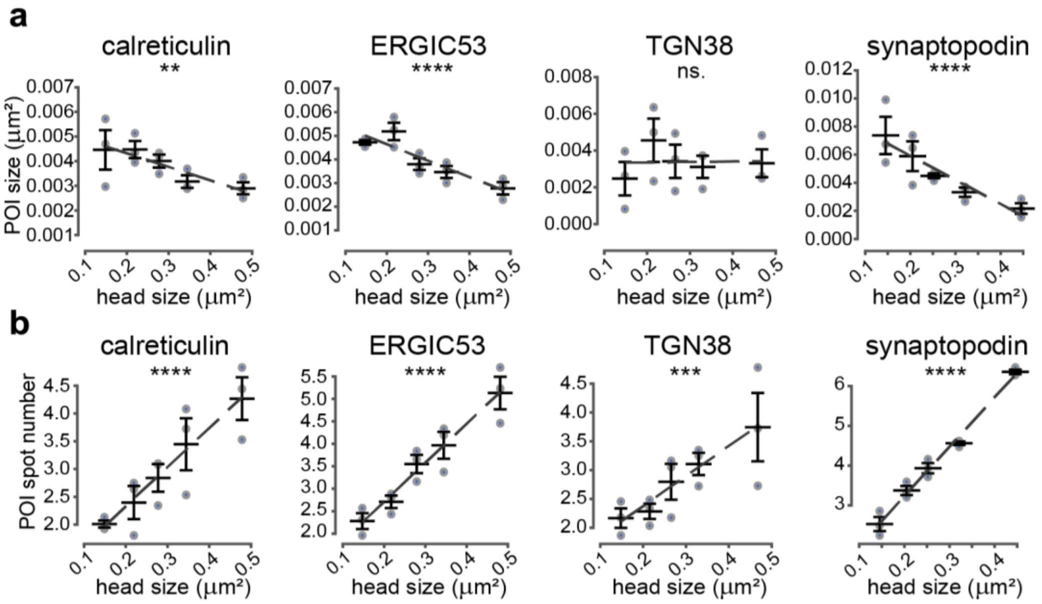

Supplementary Figure 10: Correlations of POI spot size and numbers and synaptic size in mushroom spines. (a) Correlation between average protein spot size of calreticulin, ERGIC53, TGN38, and synaptopodin inside the spine head and head size, as defined by manual segmentation. **(b)** Correlation between average spot numbers of calreticulin, ERGIC53, TGN38, and synaptopodin inside the spine head correlated to the head size as defined by manual segmentation. Dashed lines are linear fits. Where *=p<0.05, **=p<0.01, ***=p<0.001, and ****=p<0.0001. Whiskers represent S.E.M. Numbers of synapses per each bin and Spearman's rho are listed in Supplementary Table 1.

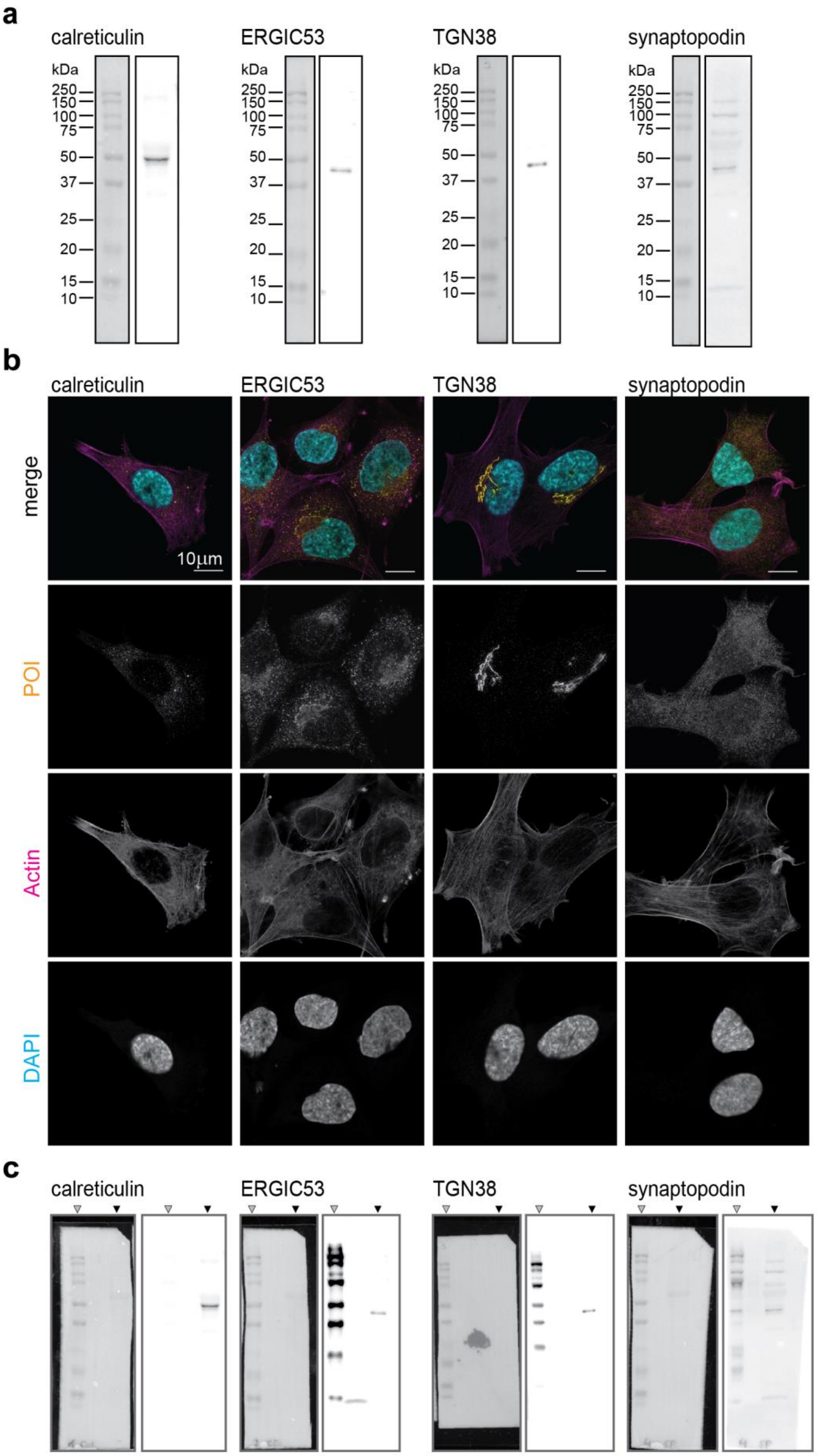
a
calreticulin
ERGIC53
TGN38
synaptopodin
kDa
250
150
100
75
50
37
25
20
15
10
b
merge
POI
Actin
DAPI
10μm
c

Supplementary Figure 11: Antibody validation. (a) Western blot validation of used POI antibodies in whole cell lysate of PC12 cells (calreticulin, ERGIC53 and synaptopodin) or rat cultured primary cortical neurons (TGN38). Displayed are colourimetric images of the protein ladder (left) and the respective antibody chemilumenescence signal (right) of each individual full-length membrane. Proteins have been blotted and analysed on separate membranes. Brightness and contrast have not been modified and are displayed as raw data. Full colourimetric and chemiluminescence images of the membranes are shown in panel (c).
(b) Representative STED images of immunofluorescence in PC12 cells labelled with DAPI (confocal), phalloidin (actin), and the proteins of interest: calreticulin, ERGIC53, TGN38, and synaptopodin. Scale bars: 10 µm. **(c)** Full size membrane images used for panel (a). Left: colourimetric; right: chemilumenescence. Grey arrows indicate the position of the protein ladder; black arrows indicate the lane of the respective cell lysate, as shown in (a). The protein ladder shows aspecific chemilumenescence signal in the case of ERGIC53, TGN38 and synaptopodin. Brightness and contrast have not been modified.

Supplementary Methods

Sample preparation and imaging of PC12 cells

PC12 adh cells (AddexBio, cat. C0032002) were cultured on glass coverslips and processed as described in the main text. Actin was labelled with phalloidin-Star635P (Abberior, cat. 2-0205-002-5; 1:100 dilution) and nucleus with DAPI (Biomol, cat. AG-CR1-3668-M005, 1:5000 dilution). Imaging was performed as previously described.

Western Blot

Whole cell lysate from PC12 adh cells or cultured cortical primary neurons (18 days *in vitro*, prepared as hippocampal cultures), in Laemmli buffer (Bio-Rad, cat. 161-0747) were loaded in a 12% SDS gel (Bio-Rad, cat. 4561045) and subjected to electrophoresis at 50 V for 5 min, followed by 10 V for 80 min. Separated proteins were then transferred onto a PVDF membrane (Bio-Rad, cat. 162-0260) at 120 V for 50 min. The blotted membranes were blocked in Tris-buffered saline, 0.1% Tween (TBST), 3% BSA and incubated overnight with previously described primary antibodies (calreticulin 1:500, ERGIC53 1:1000, TGN38 1:500, synaptopodin 1:1000) in TBST, 1% BSA. Then the membranes were washed five times in TBST and incubated with secondary antibody dilution (Thermo Fisher, cat. 31466, 1:10000) in TBST 1% BSA for 1 hour at room temperature. Afterwards membranes were washed five times in TBST, incubated for 1 min with ECL western blotting substrate (Thermo Fisher, cat. 32109) and signal was detected in a Chemidoc System (Bio-Rad Laboratories GmbH, Germany).
